# Online Visual Feedback during Error-Free Channel Trials Leads to Active Unlearning of Movement Dynamics: Evidence for Adaptation to Trajectory Prediction Errors

**DOI:** 10.3389/fnhum.2016.00472

**Published:** 2016-09-23

**Authors:** Angel Lago-Rodriguez, R. Chris Miall

**Affiliations:** School of Psychology, University of BirminghamBirmingham, UK

**Keywords:** motor adaptation, visual feedback, motor memory, trajectory prediction error, active unlearning

## Abstract

Prolonged exposure to movement perturbations leads to creation of motor memories which decay towards previous states when the perturbations are removed. However, it remains unclear whether this decay is due only to a spontaneous and passive recovery of the previous state. It has recently been reported that activation of reinforcement-based learning mechanisms delays the onset of the decay. This raises the question whether other motor learning mechanisms may also contribute to the retention and/or decay of the motor memory. Therefore, we aimed to test whether mechanisms of error-based motor adaptation are active during the decay of the motor memory. Forty-five right-handed participants performed point-to-point reaching movements under an external dynamic perturbation. We measured the expression of the motor memory through error-clamped (EC) trials, in which lateral forces constrained movements to a straight line towards the target. We found greater and faster decay of the motor memory for participants who had access to full online visual feedback during these EC trials (Cursor group), when compared with participants who had no EC feedback regarding movement trajectory (Arc group). Importantly, we did not find between-group differences in adaptation to the external perturbation. In addition, we found greater decay of the motor memory when we artificially increased feedback errors through the manipulation of visual feedback (Augmented-Error group). Our results then support the notion of an active decay of the motor memory, suggesting that adaptive mechanisms are involved in correcting for the mismatch between predicted movement trajectories and actual sensory feedback, which leads to greater and faster decay of the motor memory.

## Introduction

Mechanisms underlying human motor learning have been extensively studied by applying external visuomotor or dynamic perturbations during performance of reaching movements (for a review see Wolpert et al., 2011). Motor adaptation is an active error-based learning mechanism that relies on the updating of internal models following sensory-prediction errors (Shadmehr and Mussa-Ivaldi, 1994; Flanagan and Wing, 1997; Krakauer et al., 2000). In addition, information regarding the success or failure of a given movement drives reinforcement learning (Abe et al., 2011; Izawa and Shadmehr, 2011; Shmuelof et al., 2012; Galea et al., 2015), and use-dependent learning relies on movement repetition, which biases performance towards the repeated movement (Classen et al., 1998; Verstynen and Sabes, 2011). It has been recently proposed that motor learning may result from combining these learning processes (Huang et al., 2011), which may occur in parallel (Diedrichsen et al., 2010).

Prolonged exposure to movement perturbations leads to generation of motor memories, which can be recalled hours, and even months after being created (Shadmehr and Brashers-Krug, 1997; Joiner and Smith, 2008). Interestingly, recently created motor memories can be assessed by exposing participants to error clamped (EC) trials immediately after adaptation, in which a virtual “channel” is created by generating lateral forces that constrain the movement to a straight line towards the target; the visual feedback also reflects the straight movement. By using EC trials, we can measure the forces that participants apply towards the wall of the virtual channel because they anticipate and compensate for the perturbation that they previously experienced. It has been shown that when participants are exposed to EC trials, their motor memories decay towards previous states (Ingram et al., 2013; Vaswani and Shadmehr, 2013; Brennan and Smith, 2015; Vaswani et al., 2015). The rationale for using EC trials to evaluate the decay of the motor memory is that they are thought to disengage mechanisms of motor adaptation due to a lack of an error-signal (Smith et al., 2006). Thus decay during EC trials suggests that the process does not depend on visual feedback errors.

However, it has recently been reported that manipulating features of the EC trials may modify the decay of the motor memory—both the rate of decay, and the decay onset—(Vaswani and Shadmehr, 2013; Vaswani et al., 2015). Vaswani et al. (2015) found different patterns of motor memory decay determined by the similarity of EC trials distribution to participant’s behavior during the adaptation phase. The authors suggested that the experimental manipulation of the EC feedback led to exploratory behavior, resulting in slower decay of the motor memory because of the engagement of reinforcement-based learning processes (Vaswani et al., 2015). However, when Brennan and Smith (2015) tried to replicate Vaswani’s hypothesis of motor memory decay dependent on the detection of changes in context, the authors failed to find a significant effect of context manipulation over participants’ behavior on EC trials. Brennan and Smith (2015) showed that the decay of the motor memory started—without delay—even when context changes were masked.

Thus, there is controversy regarding whether motor memories—created based on movement errors—are intrinsically volatile and decay during EC trials, or whether there is an active motor learning process occurring during the extended EC testing of the motor memory. Therefore, we aimed to test whether motor adaptation, driven by error signals based on the sensory feedback (visual or proprioceptive) available during EC trials, contributes to the decay of the motor memory. This study evaluated whether the decay of the motor memory can be experimentally modified by manipulating online visual feedback during EC trials. We tested three groups of participants in a force-field (FF) adaptation paradigm. We measured the motor memory following adaptation using EC trials, in which there is no positional deviation of the hand, and compared the rate of decay between participants with online visual feedback of movement distance only vs. a veridical cursor, which showed the movement constrained to a straight line towards the target. We also tested a group of participants who were shown an augmented visual feedback error-signal (i.e., a cursor trajectory curved in the opposite direction to the expected movement).

## Materials and Methods

### Participants

We recruited 45 self-reported right-handed participants, who were randomly allocated to one of three experimental groups. We first recruited 30 participants, who were allocated to the Arc (mean age: 23.5 ± 1.2; 10 females) and Cursor (mean age: 23.3 ± 1.2; 10 females) groups, whereas a final group of 15 participants were allocated to the Augmented-Error group (mean age: 23.07 ± 4.5; 11 females). All participants were naive to the experimental paradigm, and the purpose of the experiment. All participants gave written informed consent in accordance with the Declaration of Helsinki, and the study was approved by the ethics committee of the University of Birmingham.

### Experimental Procedure

**Figure 1:**
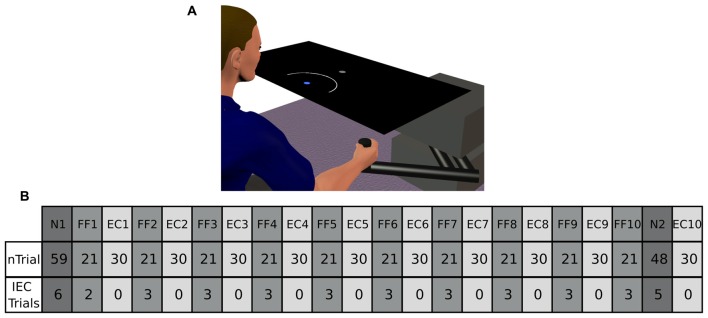
**(A)** Represents our experimental setup. Participants from the Arc group were presented an expanding arc centered on the starting position during EC and IEC Trials. The arc radius equaled the distance between the hand and the start position, thus conveying information regarding movement distance, but not regarding movement direction. The Cursor group saw a cursor identical to that shown to both groups in N and FF trials. The Augmented-Error group was presented a cursor trajectory that followed a gently curved path, in the opposite direction to the one shown by participants from Arc and Cursor groups at the end of FF blocks. **(B)** Shows a schematic representation of the main experimental protocol used in this study, which is based on protocol from Herzfeld et al. (2014). N, null-field; FF, force-field; EC, error-clamped; IEC, interspersed error-clamped. Participants performed 22 separate blocks that alternated between adaptive FF and assessment EC trials.

Participants were comfortably seated in front of a display system. They were asked to perform point-to-point reaching movements in the sagittal plane (distance 15 cm) with a 2-D robotic manipulandum (a Vbot; Howard et al., 2009). Visual feedback was displayed on a screen (refresh rate: 60 Hz), which was viewed via a mirror, so that the image was coplanar with the hand (see Figure 1A). The start and target positions were displayed as dots of 2 cm diameter. Visual feedback regarding hand position, when present, was shown as a cursor of 1 cm diameter.

Subjects were asked to hit the target within 400 ± 50 ms. Trials finished when the cursor reached the target position—so no terminal positional errors at trial end were available. Feedback regarding performance speed was given at the end of each trial. If subjects moved too fast, target and start position became blue and a message “Too Fast” was presented in the screen. Conversely, when the pointing movement was too slow, target and start position became red and a message “Too Slow” could be read on the screen. Finally, when the cursor reached the target on time, target and start position became yellow. Moreover, an explosion-like feedback was presented when subjects reached the target on time.

The aim of the task was “to hit the target on time” throughout the entire experimental session. Participants were given the opportunity to practice (192 trials in total) under non-perturbed conditions (Null-Field, N). After the familiarization period, participants performed 617 trials in total, which were divided into 22 blocks (two N blocks, 10 FF blocks, and 10 EC blocks). A scheme of the experimental design is represented in Figure 1B; and is based on the protocol reported by Herzfeld et al. (2014).

On FF trials a velocity-dependent FF was applied to the reaching movement by the robotic manipulandum. This force was proportional and orthogonal to the movement velocity. For a given movement velocity $\vec{\dot{v}}\;=\;{\left[\dot{x}\;\dot{y}\right]}^{T}$, the robot produced a force $\vec{F}$ = [*F_x_ F_y_*]^T^ equal to $\vec{F}\;=\;B\vec{\dot{v}}$, where $B\;=\;\left[\begin{array}{cc} 0 & b\\ -b & 0 \end{array}\right]$. The parameter *b* was defined as 15 N/(m/s), thus determining a clockwise curl field. During EC trials the robotic device constrained motion to a straight line towards the target by generating reactive forces (Scheidt et al., 2000). These reactive forces create a virtual spring with stiffness of 5000 N/m, and damping of 30 N/m/s in the axis orthogonal to the constrained path. Participants were also exposed to small numbers of EC trials unpredictably interspersed within blocks of N and FF trials (Interspersed EC (IEC) Trials; see Figure 1B for more details).

**Figure 2:**
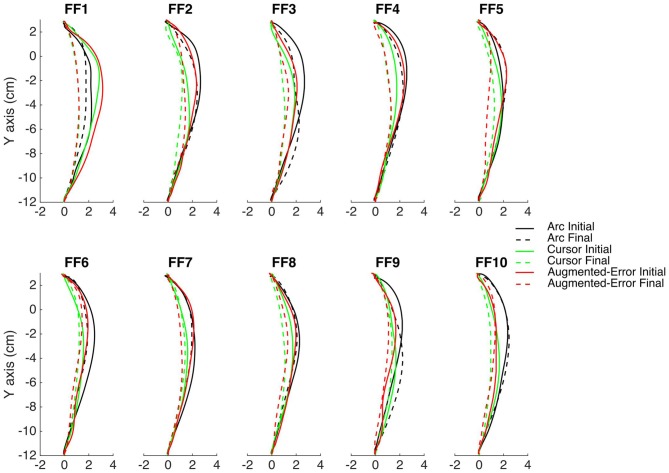
**This figure represents the average reach trajectory across participants from the experimental groups Arc, Cursor and Augmented-Error for the initial six trials (solid lines), and the last six trials (dashed lines) of each block of FF trials**.

The main experimental manipulation of this study was introduced on EC and IEC trials. Whereas participants from the Cursor group were presented an online visual cursor during EC and IEC trials, participants from the Arc group could not see the cursor and instead were presented an expanding semi-circular arc, which was centered on the start position (see Figure 1A). The radius of the arc was equivalent to the distance between the Vbot handle and the start position, thus presenting participants information regarding movement distance, although not regarding movement direction. Participants from the Augmented-Error group were presented an online visual feedback on EC blocks, which followed a gently curved path, in the opposite direction to the one shown by participants from Arc, and Cursor groups at the end of FF blocks (see dashed lines from Figure 2). This online visual feedback was position-dependent, so its forward trajectory was equivalent to the position of the participant’s hand on the *Y*-axis, whereas the position in the *X*-axis followed a circular arc. There was no lateral deviation at start or end of the movement, and the maximal lateral deviation of the cursor was 1 cm to the left of the straight line channel, at the mid-way point between the start and target positions.

### Data Collection and Analysis

Data was collected at a sample rate of 1000 Hz, and it was saved in a PC for offline analysis. Customized Matlab scripts were used to analyze each trial’s positional data.

We calculated trial-by-trial movement duration (Movement Time) as the time from movement onset to trial end. Trials were considered started when the cursor left the start position (i.e., distance between center of start position and center of cursor >2.25 cm). Trials were considered finished when the cursor reached the target (i.e., distance between cursor and target centers <0.75 cm). We then defined peak velocity, and calculated movement error as the angle (Directional Error, in degrees) between two vectors linking the position at movement onset with the target, and the position of peak velocity, respectively. For EC trials and IEC trials we measured the force participants exerted against the virtual wall at peak velocity, and normalized this force value based on peak velocity (Force at Peak Velocity).

#### Analysis of Baseline Performance

We tested for between-group differences at Baseline by performing separate univariate analysis of variance (Univariate ANOVA) for the Directional Error, Movement Time and Force at Peak Velocity collected at Baseline (N1)—when participant had not yet experienced perturbations.

#### Analysis of Motor Adaptation

In order to test for participants’ adaptation to the external dynamic perturbation along time, we performed an analysis of variance for repeated measures (ANOVA-RM) for the average within-block Directional Error, using a mixed model with Block (FF1–FF10) as a within-subject factor, and Group (Arc, Cursor, Augmented-Error) as a between-subject factor. We further explored for motor adaptation by performing an ANOVA-RM for the average within block value of the Force at Peak Velocity on IEC trials interspersed among FF trials. We used Block (FF1–FF10) as within-subject factor, and Group (Arc, Cursor, Augmented-Error) as between-subject factor. We further control for the potential interference of peak velocity on our analysis of directional error by performing an analysis of covariance (ANCOVA), with Directional Error as dependent variable, and Peak Velocity as covariate.

#### Analysis of the Motor Memory

To test for differences in the expression of motor memory, we performed an ANOVA-RM for the average within-block Force at Peak Velocity, with Block (EC1–EC9) as within-subject factor, and Group (Arc, Cursor, Augmented-Error) as between-subject factor. In order to evaluate whether manipulation of visual feedback in blocks of EC trials resulted in significant between-group differences for peak velocity, we performed a Univariate-ANOVA with Group (Arc, Cursor, Augmented-Error) as between-subject factor.

When significant between-group differences were found —either for motor adaptation or for the expression of the motor memory—we followed the analysis by calculating the average value for bins of six consecutive trials from the same experimental block. We then performed two separate ANOVA-RM, for the initial and final within block performance, respectively. This allowed us to test whether average between-group differences were driven by performance differences already evident at block onset, or alternatively by differences emerging at block end.

#### Analysis of Success Rates

Reinforcement learning has been suggested as key mechanism for protection of motor memories (Pekny et al., 2011). Since participants had to reach the target within a time window in order to successfully complete the trial and avoid a speed-warning message, we calculated the probability of being successful (i.e., not warned) based on movement duration. We considered trials successful when Movement Time was 350–450 ms. We then calculated the average within block probability of success for each experimental group. To test for potential between-group differences at baseline, we first performed a Univariate-ANOVA for the probability of success measured at N1, with Group (Arc, Cursor, Augmented-Error) as between-subject factor. We then performed an ANOVA-RM with Block (FF1–FF10) as within-subject factor, and Group (Arc, Cursor, Augmented-Error) as between-subject factor, to test for differences on the probability of success during adaptation. To test for differences on the probability of success during motor memory decay, we performed an ANOVA-RM with Block (EC1–EC9) as within-subject factor, and Group (Arc, Cursor, Augmented-Error) as between-subject factor. We finally performed a one-way ANOVA for the probability of success measured when evaluating the re-expression of the motor memory (EC10) after a washout period (N2), with Group as between-subject factor.

#### Analysis of the Motor Memory Re-Expression

To test for differences in the pattern of re-expression of the motor memory, we performed an ANOVA-RM for the average Force at Peak Velocity for bins of six consecutive trials after the washout period (EC10), with Bin (1–5) as within-subject factor and Group (Arc, Cursor, Augmented-Error) as between-subject factor. To evaluate whether participants recall previous states when re-exposed to EC trials, we performed an ANOVA-RM for the average Force at Peak Velocity at the end of blocks EC9–EC10, with Block as within-subject factor and Group (Arc, Cursor, Augmented-Error) as between-subject factor.

For all ANOVA-RM, *post hoc* comparisons were performed with Bonferroni corrections when a significant result was found. Greenhouse-Geisser corrections were performed when data violated the assumption of sphericity (fractional degrees of freedom for *F*-values are shown accordingly). A *p*-value ≤ 0.05 was considered significant for all tests. We performed the statistical analysis with the Statistical Package for the Social Sciences (SPSS; version 22.0).

#### Estimation of Rates of Decay

We estimated the rate of decay of the motor memory by fitting a single exponential model of the form y = a*e^b*x^ + c to the data within each EC block. Because fitting curves to individual participants’ data can be unstable, we performed a sub-sampling bootstrap analysis (Politis et al., 1999; in Ingram et al., 2013). Specifically, the 15 participants from each group were used to generate three separate subsets of 455 unique sub-samples (i.e., 455 subsets per each experimental group), each consisting of 12 subjects. The single exponential model was then fit to the across-participants average data series of the 30 EC trials for each of the 455 unique sub-sets. The between-group difference for the initial state parameter *a*, the decay rate parameter *b* (i.e., rate of change of the exponential curve) and the curve asymptote parameter c were then calculated for each possible pair-wise group comparison. Furthermore, we performed a permutation test on each of the 455 bootstrap iterations, for each of the three pair-wise analyses. We randomly assigned each of the 24 participants selected for each iteration to one of two experimental groups (pair-wise, comparing Arc, Cursor, Augmented-Error). We then fit the average trial-by-trial data for each new group with a single exponential model, and calculated the difference between groups for the three parameters. We then calculated the proportion of permutation samples larger than the mean difference found for the bootstrap procedure, as a measure of how likely it would be to find our results in a population where the two analyzed groups did not differ.

## Results

### Baseline Performance

To test for between-group differences at Baseline (N1), we performed three separate Univariate ANOVAs for the Directional Error, Movement Time and Force at Peak Velocity, respectively. We did not find significant between-group differences for Directional Error (*F*_(2,42)_ = 1.33; *p* = 0.27), or Force at Peak Velocity (*F*_(2,42)_ = 0.39; *p* = 0.68). We found significant between-group differences for Movement Time (*F*_(2,42)_ = 35.41; *p* < 0.001), and *post hoc* comparisons revealed that participants from the Augmented-Error group performed the reaching task significantly slower at baseline than participants from both the Cursor and Arc groups (*p* < 0.001 for both comparisons; see Figure 5). As we show later, this difference was not maintained during the adaptation phase, and it did not have an effect on the rate of success (i.e., it would not influence reinforcement learning).

### Motor Adaptation

#### Directional Error at Peak Velocity

**Figure 3:**
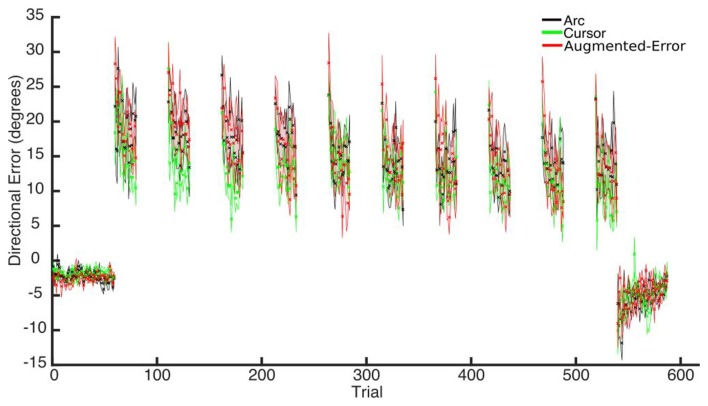
**Across participants average trial-by-trial directional error (degrees) measured at blocks of N (N1, N2) and FF (FF1–FF10) trials.** Values are shown as mean ± SEM.

**Figure 4:**
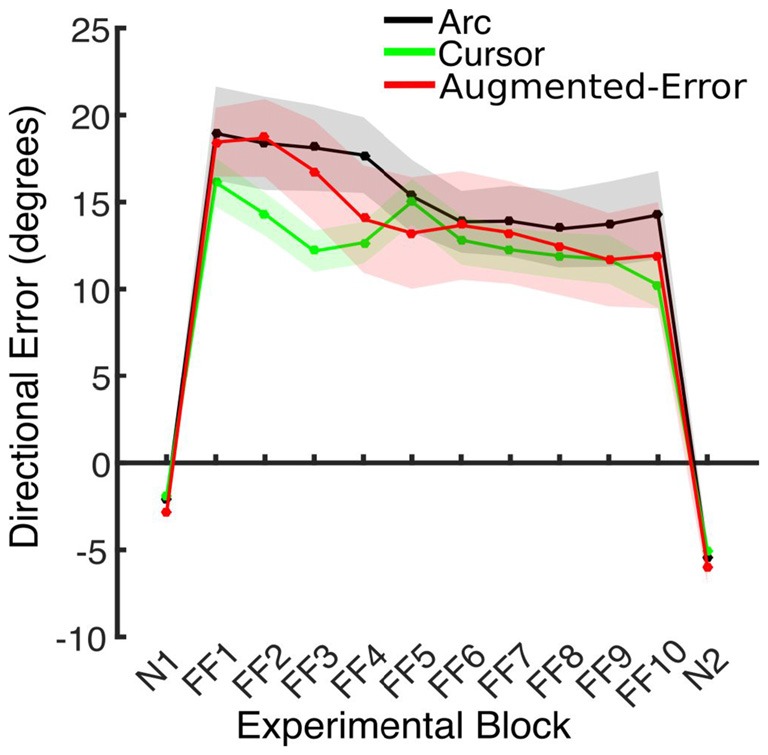
**This figure represents the absolute directional error shown by participants at peak velocity.** Values are represented as mean ± SEM.

Trial-by-trial average directional error shown by participants at N and FF blocks is presented in Figure 3. An ANOVA-RM for the average within-block directional error measured during FF blocks showed a significant effect for Block (FF1–FF10: *F*_(2.6,110.8)_ = 7.91; *p* < 0.001; Figure 4). We did not find a significant effect for Group (Arc, Cursor, Augmented-Error: *F*_(2,42)_ = 0.55; *p* = 0.58), or a significant Block × Group interaction (*F*_(5.3,110.8)_ = 0.94; *p* = 0.46).

We further analyzed motor adaptation by calculating the average value for the initial and final performance shown at each FF block (values were calculated based on bins of six consecutive trials). An ANOVA-RM for the initial performance revealed a significant effect for Block (FF1–FF10: *F*_(3.7,155.9)_ = 7.69; *p* < 0.001). However, we did not find significant effect for Group (*F*_(2,42)_ = 0.47; *p* = 0.63), or significant Block × Group interaction (*F*_(7.4,155.9)_ = 0.79; *p* = 0.60). An ANOVA-RM for the performance showed by participants at the end of FF blocks revealed a significant effect for Block (*F*_(4.5,188.5)_ = 4.95; *p* < 0.001). We did not find significant effect for Group (*F*_(2,42)_ = 1.14; *p* = 0.33), or significant Block × Group interaction (*F*_(9.0,188.5)_ = 0.96; *p* = 0.48). Thus participants showed significant adaptation to the perturbation forces, but in all measures the FF performance of the three groups was similar.

We also control for the potential influence of peak velocity on our analysis of directional error by performing an ANCOVA, with Directional Error as dependent variable, and Peak Velocity as covariate. We found a significant effect of Block (*F*_(9,419)_ = 2.64; *p* = 0.006) and Group (*F*_(2,419)_ = 4.59; *p* = 0.011) factors. We did not find a significant Block × Group interaction (*F*_(18,419)_ = 0.31; *p* = 0.997). *Post hoc* comparisons revealed a significantly smaller directional error for the Cursor group, compared both with the Arc (*p* = 0.018) and Augmented-Error (*p* = 0.03) groups.

#### Movement Time

**Figure 5:**
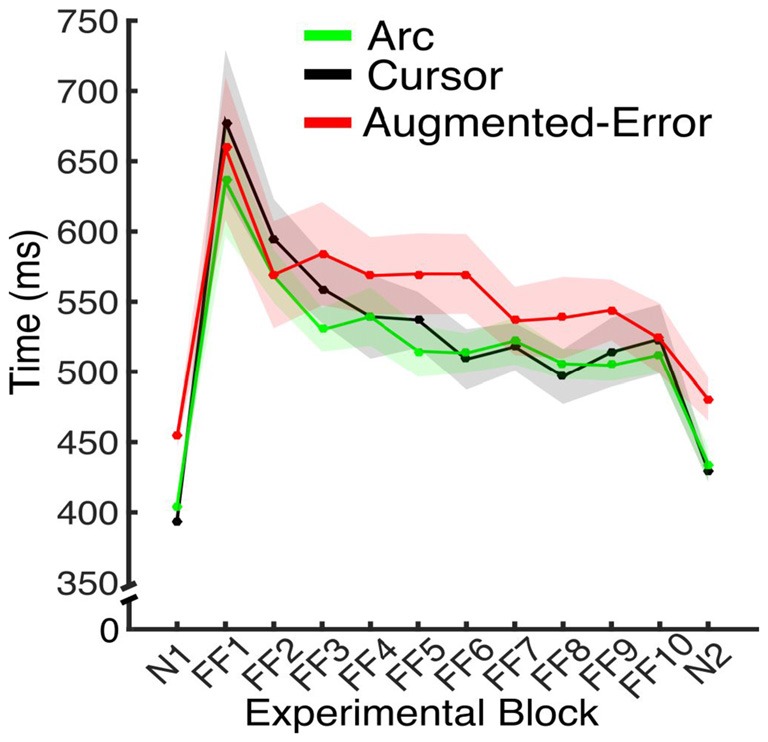
**Average movement time for the N and FF blocks, for each experimental group.** Values are shown as mean ± SEM.

Since feedback about performance success was determined by movement time (see “Materials and Methods” Section), subjects had to adjust to changes in movement time resulting from introduction of the dynamic perturbation. Thus, we performed an ANOVA-RM for movement duration, with Block (FF1–FF10) as within-subject factor, and Group (Arc, Cursor, Augmented-Error) as between-subject factor. Results showed a significant effect for Block (*F*_(3.6,151.0)_ = 20.29; *p* < 0.001; Figure 5). However, we did not find an effect for Group (*F*_(2,42)_ = 1.91; *p* = 0.16), or a significant Block × Group interaction (*F*_(7.2,151.0)_ = 0.81; *p* = 0.59). Thus, all three groups equally adapted their movements on the time dimension when the perturbation was introduced, although they were not fully successful in complying with the time constraints.

We further analyzed movement duration by calculating the average value for the initial and final performance from each FF block (values were calculated based on bins of six consecutive trials). An ANOVA-RM for the initial performance revealed a significant effect for Block (FF1–FF10: *F*_(2.9,122.5)_ = 23.04; *p* < 0.001). However, we did not find significant effect for Group (Arc, Cursor, Augmented-Error: *F*_(2,42)_ = 1.16; *p* = 0.32), or significant Block × Group interaction (*F*_(5.8,122.5)_ = 0.94; *p* = 0.47). An ANOVA-RM for the performance showed by participants at the end of FF blocks revealed a significant effect for Block (*F*_(6.9,290)_ = 3.42; *p* = 0.002), and a significant Block × Group interaction (*F*_(13.8,290.0)_ = 1.76; *p* = 0.044). We did not find significant effect of Group (*F*_(2,42)_ = 0.52; *p* = 0.6). Pair-wise *post hoc* comparisons revealed significant difference when movement time from the Arc group at FF8 was compared with FF1 (*p* = 0.006) and FF2 (*p* = 0.031), and when time at FF2 was compared with FF7 (*p* = 0.049). However, the analysis did not reveal any between-group significant differences along each of the 10 blocks of practice (FF1–FF10).

#### Force at Peak Velocity in Interspersed EC Trials

**Figure 6:**
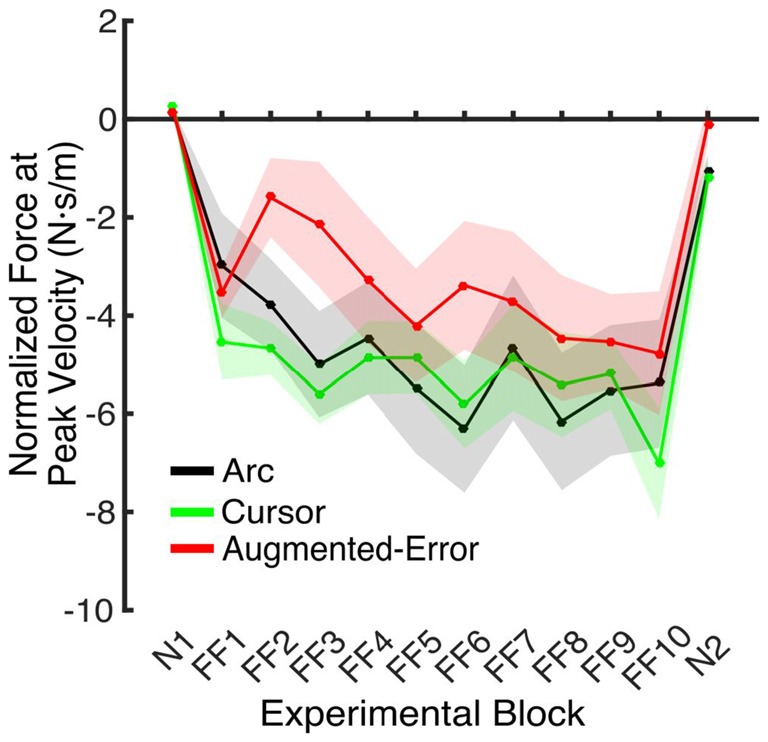
**Force applied at peak velocity in IEC trials (e.g., EC trials introduced within a N and a FF block).** Values are represented as across trials average mean ± SEM.

An ANOVA-RM for the anticipatory lateral force exerted by participants at peak velocity in IEC trials (e.g., EC trials interspersed within blocks of FF trials) revealed a significant effect for Block (*F*_(4.5,189.1)_ = 4.2, *p* = 0.002; Figure 6). We did not find an effect for Group (*F*_(2,42)_ = 1.0, *p* = 0.38), or a Block × Group interaction (*F*_(9.0,189.1)_ = 1.18, *p* = 0.31).

**Figure 7:**
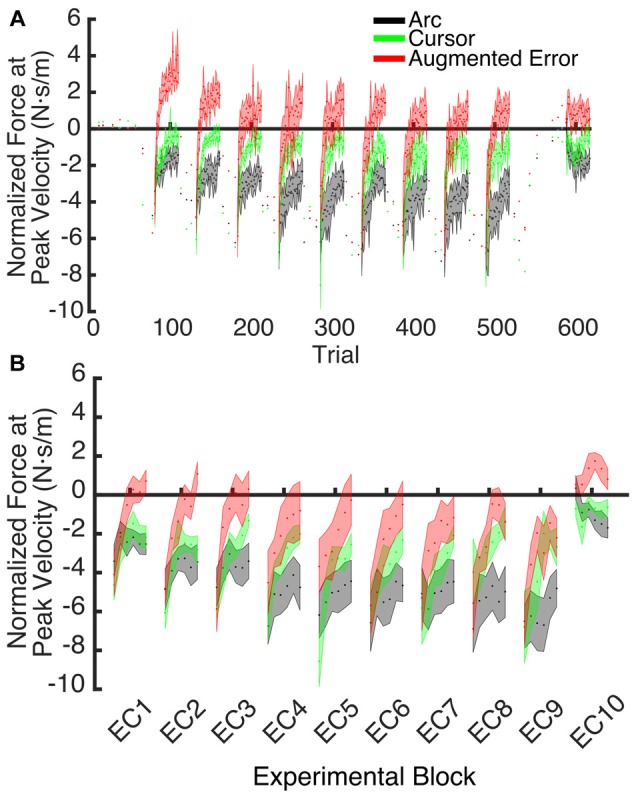
**(A)** Shows the trial-by-trial Force exerted by participants at peak velocity on EC and IEC trials. Values are shown as mean ± SEM; **(B)** Represents the trial-wise Force at Peak Velocity assessed on the initial six trials of blocks of EC trials. Values are shown as mean ± SEM.

### Expression of the Motor Memory

**Figure 8:**
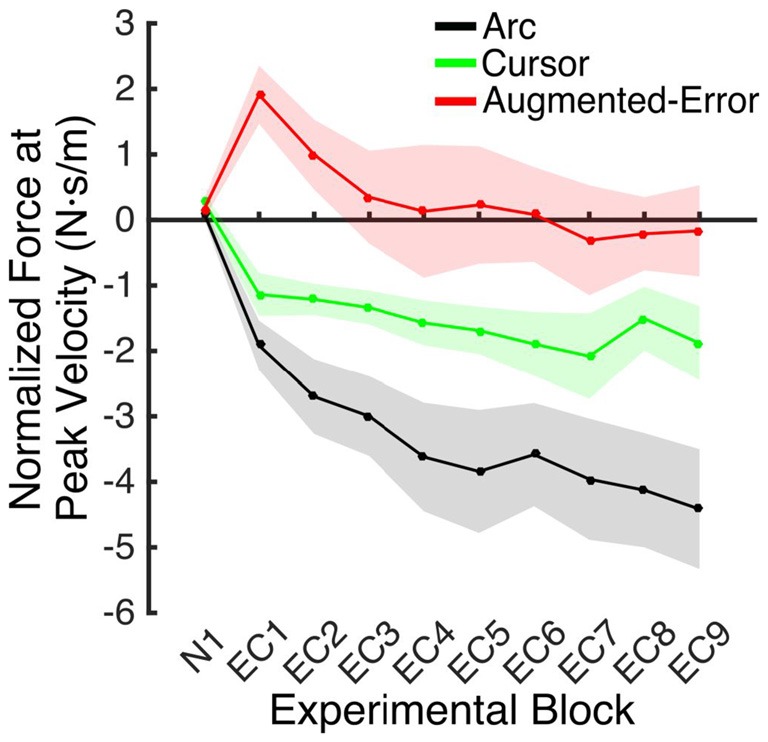
**Mean development of the motor memory calculated as the average force applied at peak velocity at baseline (N1), and at blocks of EC trials (EC1–EC9) following adaptation.** Values are shown as mean ± SEM.

An ANOVA-RM for the mean Force exerted at Peak Velocity during blocks of EC trials (EC1–EC9) revealed significant effect for Block (*F*_(3.1,130.8)_ = 8.55; *p* < 0.001), and a significant effect for Group (*F*_(2,42)_ = 10.61; *p* < 0.001). We did not find significant Block × Group interaction (*F*_(6.2,130.8)_ = 0.87; *p* = 0.52). *Post hoc* comparisons revealed significant differences between the Augmented-Error (0.3 ± 0.6 N·s·m^−1^) and Arc (−3.5 ± 0.6 N·s·m^−1^) groups (*p* < 0.001), and a trend for significance was found when we compared the Augmented-Error with the Cursor (−1.6 ± 0.6 N·s·m^−1^) group (*p* = 0.073), as well as for the comparison between Cursor and Arc groups (*p* = 0.085). Thus, these results suggest a differential expression of the motor memory for the Augmented-Error group, with the Augmented-Error group shifting their mean responses to oppose the curvature of the cursor’s trajectory (Figure 8).

**Figure 9:**
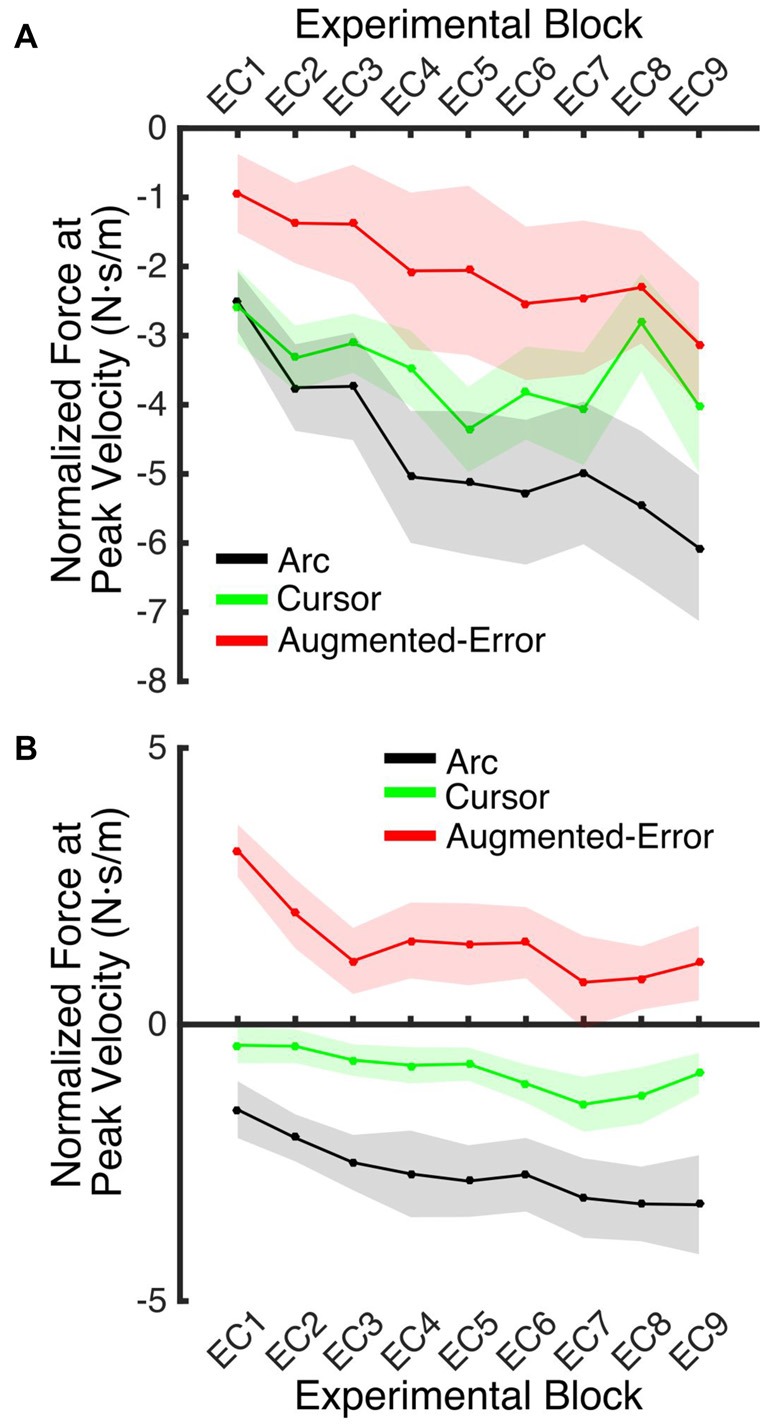
**(A)** Represents participants’ initial performance at blocks of EC trials. **(B)** Represents the average final performance on EC blocks. Results were calculated as average of six consecutive trials. Values are shown as mean ± SEM.

We next analyzed the pattern of change of the motor memory within blocks of EC trials by calculating the average value for the initial and final performance within each block (values were calculated based on bins of six consecutive trials). An ANOVA-RM for the initial performance revealed a significant effect for Block (EC1–EC9: *F*_(3.8,160.2)_ = 6.56; *p* = < 0.001; Figure 9A), and Group (*F*_(2,42)_ = 3.25; *p* = 0.049) factors. We did not find significant Block × Group interaction (*F*_(7.6,160.2)_ = 0.85; *p* = 0.56). *Post hoc* comparisons revealed significant differences between Augmented-Error (−2.02 ± 0.7 N·s·m^−1^) and Arc (−4.66 ± 0.7 N·s·m^−1^) groups (*p* = 0.044). An ANOVA-RM for the performance showed by participants at the end of the EC blocks revealed both a significant effect for Block (*F*_(4.8,202.7)_ = 6.46; *p* < 0.001) and Group (*F*_(2,42)_ = 18.54; *p* < 0.001; Figure 9B). We did not find a significant Block × Group interaction (*F*_(9.6,202.7)_ = 0.57; *p* = 0.83). *Post hoc* comparisons revealed that participants from the Augmented-Error (1.49 ± 0.5 N·s·m^−1^) group showed lesser expression of the motor memory, when compared both with the Arc (−2.66 ± 0.5 N·s·m^−1^) and Cursor (−0.84 ± 0.5 N·s·m^−1^) groups (*p* < 0.001 and *p* = 0.004, respectively). Moreover, we found significant differences for the motor memory expressed at the end of EC blocks between the Cursor and Arc groups (*p* = 0.033).

Since we did not expect to find between-group differences at the onset of blocks of EC trials, we also analyzed the pattern of change of the motor memory within blocks of EC trials by calculating the average value of the initial three consecutive trials within each EC block. An ANOVA-RM revealed a significant effect for Block (EC1–EC9: *F*_(4.8,200.1)_ = 5.40; *p* < 0.001). However, we did not find significant effect of Group (*F*_(2,42)_ = 1.23; *p* = 0.30), or a significant Block × Group interaction (*F*_(9.5,200.1)_ = 0.81; *p* = 0.61).

These results suggest that the initial retention of the motor memory was similar for all three groups on entry to each EC block, but that the Augmented-Error group retention was most rapidly lost, presumably affected by the change in cursor trajectory (Figure 9A). Note that adaptation in the interleaved FF blocks was equivalent for all three groups (Figure 6), but the groups increasingly deviated across the EC blocks (Figure 9B). Conversely, the Cursor group performance in EC block systematically changed across the experiment, suggesting that the rate of active unlearning of the motor memory was coupled to the amount of adaptation to the external perturbation (see Figure 7B).

In order to further test whether between-group differences found across blocks EC1–EC9 resulted from subtle learning differences, we performed an ANCOVA, with Force at Peak Velocity averaged across the last six trials of each block as dependent measure, and Force at Peak Velocity measured at the initial six trials as covariate. We found a significant effect for Group (*F*_(2,419)_ = 98.05; *p* < 0.001) and Block (*F*_(9,419)_ = 4.17; *p* < 0.001) factors, but we did not find a significant Block × Group interaction (*F*_(18,419)_ = 0.82; *p* = 0.68). *Post hoc* comparisons revealed significant differences for all pair-wise comparisons for the Group factor (*p* < 0.001 for all pair-wise comparisons). The Arc group (−2.00 ± 0.14 N·s·m^−1^) showed the most negative value of Force at Peak Velocity, followed by the Cursor (−0.71 ± 0.13 N·s·m^−1^) and Augmented-Error (0.77 ± 0.14 N·s·m^−1^) groups. These results then support the notion that between-group differences for the motor memory decay arose mainly during the blocks of EC trials, and that are not just due to potential between-group differences for learning.

**Figure 10:**
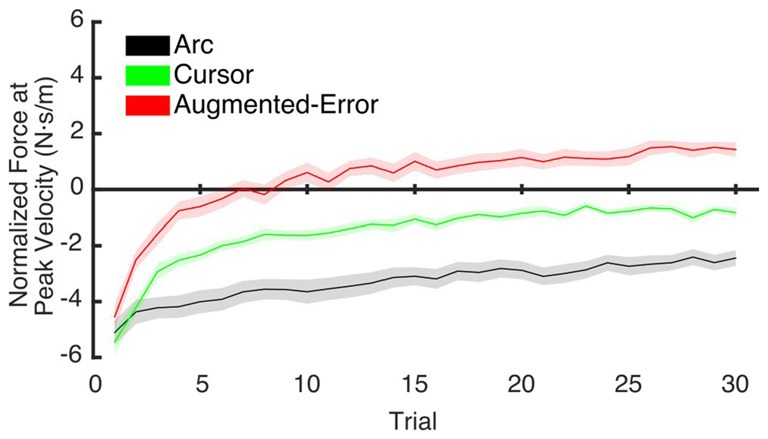
**This figure represents data resulting from the bootstrap procedure performed to estimate the rate of decay of the motor memory.** It shows the average trial-by-trial values for the Force exerted at Peak Velocity, averaged across blocks 1–9 of EC trials. Values are shown as mean ± SD.

To test for potential between-group differences for the rate of decay of the motor memory, we calculated the trial-by-trial Force at Peak Velocity, averaged for each participant across nine blocks of EC trials that followed adaptation (EC1–EC9; Figure 10). Since fitting individual data with a single exponential model can be is unstable, we performed a sub-sampling bootstrap for each combination of two experimental groups (pair-wise comparison of Arc, Cursor, and Augmented-Error) followed by a permutation test randomly reassigning every participant to one of two groups. This analysis gave reliable exponential fits, with a minimum *r^2^* value of 0.89 (mean = 0.95, and SD = 0.01). A summary of the results from the exponential fit is presented in Table 1. We did not find significant between-group differences for the initial state, suggesting that all groups had similar motor memory at its decay onset. Results found for the rate of decay (*b* parameter) revealed that the Cursor (−0.26 ± 0.02) and Augmented-Error (−0.25 ± 0.03) groups showed a rate of motor memory decay significantly larger than the Arc group (−0.08 ± 0.02). Results found for the asymptote (*c* parameter) revealed that the Arc group (−2.27 ± 0.23) showed a plateau value significantly smaller than both the Cursor (−0.90 ± 0.16) and Augmented-Error (1.15 ± 0.27) groups, whereas the Cursor group showed a plateau value significantly smaller than the Augmented-Error group.

**Table 1:** **Summary of the results revealed by the pair-wise comparison of exponential fits**.

Pair-wise comparison	*p*-value initial state *(a + c* parameter)	*p*-value rate of decay (*b* parameter)	*p*-value curve asymptote (*c* parameter)
Arc vs. cursor	0.1206	**0.0219**	**0.0285**
Arc vs. augmented-error	0.3092	**0.0219**	**0.0022**
Cursor vs. augmented-error	0.2500	0.4474	**0.0066**

*Significant values (p < 0.05) are in bold*.

### Reinforcement-Based Learning

#### Probability of Success

**Figure 11:**
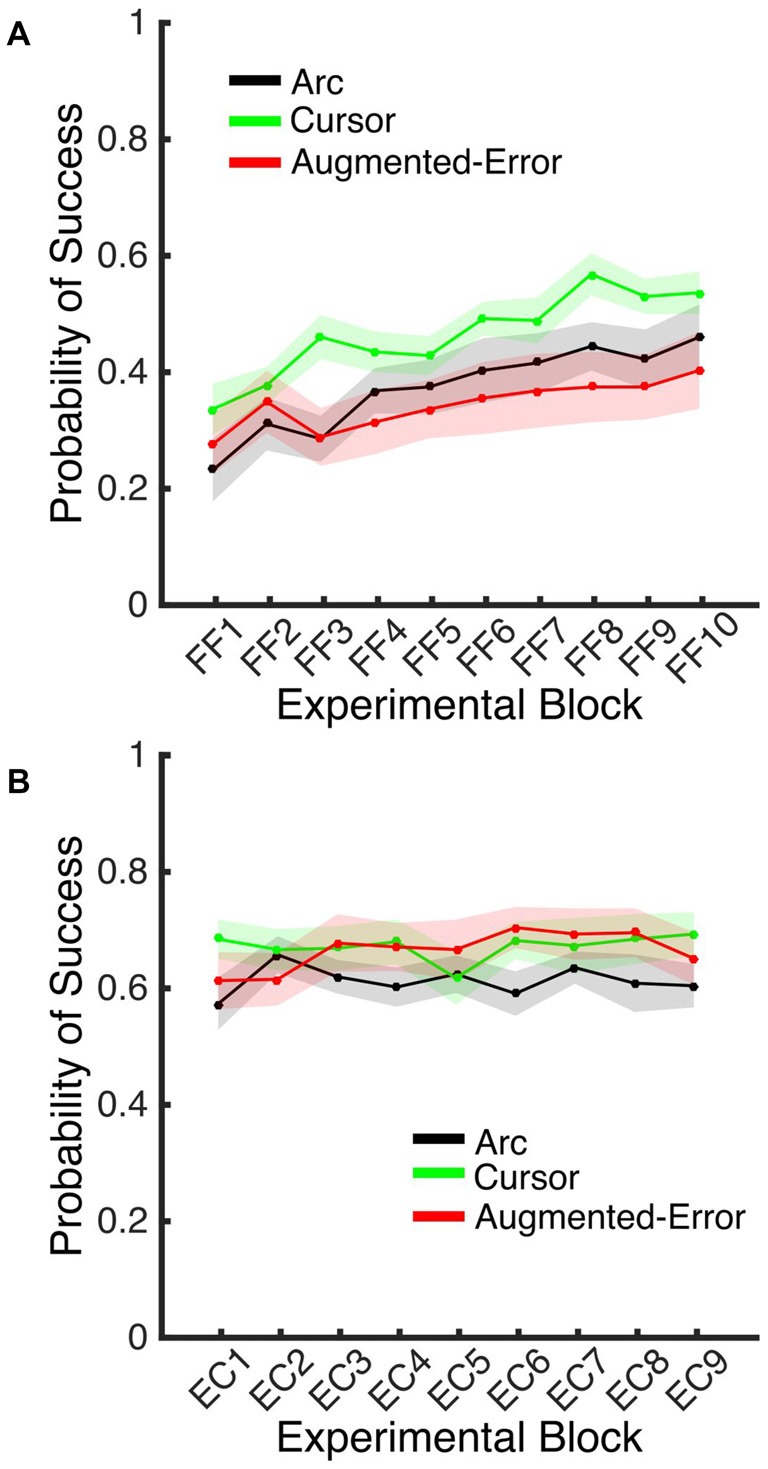
**(A)** Represents the average probability of success on blocks of FF trials. **(B)** Represents the average probability of success on EC blocks that followed adaptation (EC1–EC9). Trials were considered successful when movement duration was 350–450 ms. All Values are shown as mean ± SEM.

A Univariate ANOVA for the Probability of Success achieved by participants at Baseline did not reveal a significant effect for Group (*F*_(2,42)_ = 0.1; *p* = 0.90). However, an ANOVA-RM for the adaptation phase, Block (FF1–FF10) and Group, revealed a significant effect for Block (*F*_(9,378)_ = 12.77, *p* < 0.001), and a trend for an effect of Group factor (*F*_(2,42)_ = 2.48, *p* = 0.096; Figure 11A). We did not find a significant Block × Group interaction (*F*_(18,378)_ = 1.10, *p* = 0.35). An ANOVA-RM for Block (EC1–EC9) and Group revealed no significant effect for Block (*F*_(8,336)_ = 0.64, *p* = 0.74) or Group (*F*_(2,42)_ = 1.11, *p* = 0.34), and no significant Block × Group interaction (*F*_(16,336)_ = 0.96, *p* = 0.50; Figure 11B). A one-way ANOVA for the probability of success measured at EC10 revealed no between-group differences (Arc: 0.70 ± 0.17, Cursor: 0.70 ± 0.17, Augmented-Error: 0.71 ± 0.17; *F*_(2,42)_ = 0.036, *p* = 0.96).

#### Exploratory Behavior

In order to test for between-group exploratory behavior differences, we analyzed the variance of the directional angle during FF (FF1–FF10) blocks. We did not find significant effect for Block (*F*_(5.9,247.3)_ = 1.41; *p* = 0.21) or Group (*F*_(2,42)_ = 0.04; *p* = 0.96), and no Block × Group interaction was found (*F*_(11.8,247.3)_ = 1.0; *p* = 0.45). We next performed an ANOVA-RM for the within block average variance of the Force applied at Peak Velocity in EC1–EC9. We found a trend for a significant effect for Block (*F*_(5.2,216.7)_ = 2.07; *p* = 0.068), and Group (*F*_(2,42)_ = 3.07; *p* = 0.057) factors. However, we did not find a significant Block × Group interaction (*F*_(10.3,216.7)_ = 0.76; *p* = 0.68). *Post hoc* comparisons revealed only a trend for significant differences between Arc (1.67 ± 0.22 N·s·m^−1^) and Augmented-Error (2.43 ± 0.22 N·s·m^−1^) groups (*p* = 0.06).

Altogether, these results then suggest that the between-group difference found for the decay of the motor memory did not result from differences in engagement of reinforcement-based learning mechanisms. We did not find between-group difference for the probability of success, or for the behavioral variance (e.g., exploratory behavior) either within blocks of FF or EC trials.

### Recall of the Motor Memory

**Figure 12:**
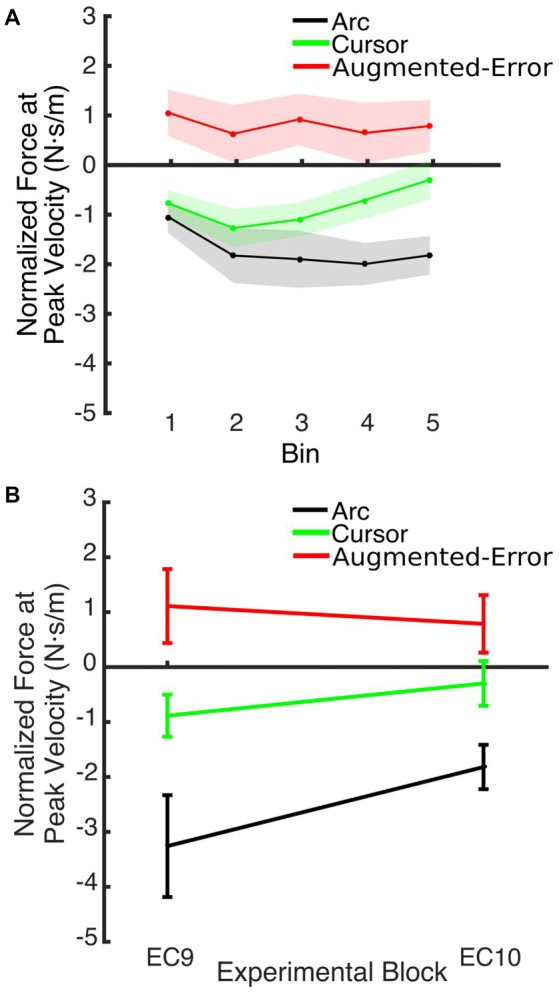
**(A)** Shows the smoothed (bins of six trials) Force at Peak Velocity, measured at recall (during block EC10). **(B)** Shows that between-group differences for the Force exerted at Peak Velocity found at the end of EC9 remained significant at the end of EC10. Values are shown as mean ± SEM.

An ANOVA-RM for the Force at Peak Velocity measured at EC10, after the washout block N2, with Time (Initial vs. Final set of six trials) as within-subject factor and Group as between-subject factor, revealed a significant effect for Group (*F*_(2,42)_ = 9.59; *p* < 0.001), and a trend for a significant effect of Time (*F*_(3.1,129.7)_ = 2.24; *p* = 0.085). However, we did not find significant Time × Group interaction (*F*_(6.2,129.7)_ = 1.35; *p* = 0.24). *Post hoc* comparisons revealed that the force exerted at peak velocity by participants from the Augmented-Error group was significantly larger, when compared both with the Arc and Cursor groups (*p* < 0.001, and *p* = 0.019, respectively; see Figure 12). Moreover, an ANOVA-RM for the Force at Peak Velocity exerted at the end of blocks EC9–EC10 revealed a significant effect for Group (*F*_(2,42)_ = 11.58; *p* < 0.001), and a trend for a significant effect of Block (*F*_(1,42)_ = 3.01; *p* = 0.09) although we did not find a Block × Group interaction (*F*_(2,42)_ = 2.42; *p* = 0.101). *Post hoc* comparisons revealed that the force exerted by the Arc group was significantly more negative compared with the Cursor and Augmented-Error groups (*p* = 0.03 and *p* < 0.001, respectively). Altogether, these results suggest that when re-exposed to EC trials, all three groups similarly recalled the motor memory that they had generated during adaptation (FF1–10), and had previously expressed at EC9.

## Discussion

In this study, we aimed to test whether mechanisms of active motor adaptation may contribute to the assumed passive decay of motor memory, by manipulating available visual feedback. The use of EC trials, which are experimentally manipulated so the movement is constrained by a channel to be in a straight line towards the target (Scheidt et al., 2000), has been extensively reported in the literature for evaluation of the motor memory (Smith et al., 2006; Vaswani and Shadmehr, 2013; Brennan and Smith, 2015). Thus, we asked participants to adapt to an external dynamic perturbation, and then measured the motor memory through EC trials. We tested three experimental groups, and introduced our main experimental manipulation during EC trials (both EC and IEC trials). One group of participants (Cursor) were presented with a cursor, which gave them full access to online visual feedback regarding hand position—constrained, like the movement itself, to be in a straight line towards the target. For the second group (Arc), we reduced the online visual feedback by presenting them with an expanding arc centered on the start position. The radius of the arc therefore provided feedback regarding movement distance, but no directional information. For the final third group (Augmented-Error), we artificially increased the visual error signal by showing participants a trajectory curved in the opposite direction to the expected path of their movements on non-clamped trials.

We found that greater mismatch between the expected trajectory and the actual visual information regarding hand position led to larger and faster decay of the motor memory. Participants from Augmented-Error and Cursor groups retained less motor memory than participants from Arc group at the end of each EC block (EC1–EC9) that followed adaptation in the FF blocks (Figure 9B), and our analysis suggests that these differences were driven by a faster rate of decay within the EC block. Moreover, participants from the Augmented-Error group showed greater decay of the motor memory, compared with the Cursor group. Importantly, differences in the decay of motor memory were not accompanied by significant between-group differences in the ability to adapt to the external dynamic perturbation. When evaluating participants’ behavior in the FF blocks, we only found significant between-group differences when we corrected values of the directional error by peak velocity. This analysis revealed that the Cursor group showed significant smaller corrected directional errors than the Arc and Augmented-Error groups. However, if this difference in directional errors was a causal factor for our finding regarding the motor memory decay, the similar errors between the Arc and Augmented-Error groups would predict similar decay rates, which is inconsistent with our findings. Thus, despite visual feedback differences on IEC trials—the EC trials randomly interspersed among each block of 18 FF trials—all groups showed similar levels of adaptation to the imposed perturbations. Altogether, these results suggest that manipulating the online visual feedback available on EC trials leads to significant changes on the rate of decay of the motor memory, although it had no effect over the ability to adapt to movement perturbation.

A relationship between reinforcement learning mechanisms and stronger motor memories has previously been proposed (Huang et al., 2011; Shmuelof et al., 2012). Moreover, Izawa and Shadmehr (2011) proposed that degrading the quality of sensory feedback led learners to switch from error-based learning to a reinforcement-based learning mechanism. This is in line with recent reports of slower decay of the motor memory when exploratory adaptive behavior had been triggered by exposing participants to error clamp trials with non-zero error and zero variance (Vaswani et al., 2015). Thus, it may be possible that modification of visual feedback on EC trials for the Arc group engaged mechanisms of reinforcement-based learning, resulting in slower decay of the motor memory. In order to discount this explanation, we evaluated both the probability of success, and the behavioral variance (i.e., exploratory behavior) for blocks of EC trials. We did not find significant between-group differences, which suggest that differences in the decay of the motor memory did not result from activation of reinforcement-learning mechanisms in the Arc group. Therefore, our results support the notion of active motor learning during the decay of the motor memory (Vaswani and Shadmehr, 2013; Vaswani et al., 2015), although they suggest a contribution of a learning mechanism different from reinforcement-based learning.

We suggest instead that error-based motor adaptation—based on sensory-prediction errors—allowed participants from the Cursor and Augmented-Error groups to actively change their motor memory more, and faster, than participants from the Arc group. Crucially, sensory-prediction errors used by Cursor and Augmented-Error groups could not be based on positional errors at trial end, since EC trials were specifically designed to avoid such errors. Thus, we propose that participants used sensory-prediction errors based on an internal error signal arising from the difference between predicted and actual movement trajectories (see Figure 57.1 in Hardwick et al., 2013). When in an “adapted” state, the motor system would predict non-straight trajectories, which for the Cursor and Augmented-Error groups would lead to a mismatch between predicted and actual sensory feedback during EC blocks. This mismatch would result in changes of upcoming motor commands. In contrast, the Arc group received no directional feedback, and thus the slower change in their performance in EC blocks would reflect a non-error-based decay. This hypothesis is further supported by our results found during adaptation to perturbations. Participants from all three groups must have adapted to the external dynamic perturbation by using trajectory errors, rather than terminal errors, since positional errors at trial end were not allowed—note that participants had to reach the target in order to finish the trial. Thus, our results suggest that mechanisms of motor adaptation were active both during adaptation to perturbations and during the decay of the motor memory, and these mechanisms used sensory-prediction errors—error signals arising from comparing predicted movement trajectories with actual sensory feedback arriving from the periphery to the central nervous system—in order to modify upcoming motor commands. This is in line with the notion of active motor learning occurring during the decay of the motor memory (Vaswani and Shadmehr, 2013; Vaswani et al., 2015), suggesting that this decay can be actively manipulated not only by engaging mechanisms of reinforcement-based learning, but also by engaging mechanisms of error-based motor adaptation.

Our theory hence predicts that artificially manipulating the available error signal during EC trials would modulate the rate of decay of the motor memory. Presenting subjects with a movement trajectory close to the trajectory expected by the motor system should lead to lesser decay, whereas an augmented error signal (i.e., movement trajectory directed towards the same direction than the perturbation previously experienced) should result in higher rate or magnitude of decay of the motor memory. Crucially, when we increased the error signal by showing participants a trajectory curved to the opposite direction than the expected path (Augmented-Error group), we found significant differences for the rate of decay, compared with the Arc group, and we also found between-group differences for the maximum value of the motor memory at the end of EC blocks, when compared both with the Cursor and Arc groups. Forces applied by participant from the Augmented-Error group aimed towards the opposite direction than the curved visual feedback trajectory (i.e., participants tried to correct the visual error). These results confirm our suggestion, that the decay of the motor memory may be driven by active mechanisms of motor adaptation, where movement corrections depend on a mismatch between expected and actual visual feedback.

We hypothesize that the sensory prediction error causes active unlearning. For the Arc group, we deny them access to visual feedback in the EC trials, and as the compensatory forces build up over EC1–9 (see Figures 6, 9A), there is relatively little change in the unlearning across the experiment (Figure 7B). In contrast, for the Cursor group, the sensory prediction error increases as learning builds up, because they do have access to visual feedback, and so can derive the error. Hence the unlearning slope over trials 1–6 steadily increases from a level initially similar to Arc during EC1 (because there is limited change in their sensory prediction) to a level in EC9 equal to that of the Augmented Error group. Finally, the Augmented Error group show fast early unlearning even in EC1 because even without any change in their sensory predictions, they experience a prediction error from the distorted visual feedback. This active unlearning rate is maintained throughout EC2–9. However, we failed to find between-group differences for the rate of motor memory decay when we compared the Augmented-Error and Cursor groups (parameter *b*, Table 1), despite our expectation that the prediction error in the Augmented-Error group should be larger. We suggest therefore that Figure 7, and particularly Figure 7B shows evidence of a monotonic relationship between sensory prediction error and decay of the forces across EC blocks, but this is probably a relationship that saturates at higher levels—there is an upper limit to the unlearning rate—and further increase of the sensory prediction error has less and less effect on unlearning rate. This would further explain the lack of between-group differences for the rate of motor memory decay when we compared the Augmented-Error and Cursor groups, and leads to future studies aimed to understand the relationship between the size of the sensory-prediction error against the unlearning rate.

Several theories have been proposed regarding the mechanisms that govern the decay of the motor memory. It may be that the decay of the motor memory results from a process that minimizes a cost function by reducing both the kinematic error (i.e., trajectory errors) and the effort (i.e., muscle activation; Emken et al., 2007; Ganesh et al., 2010). Based on a two-state space model of motor learning proposed by Smith et al. (2006), the spontaneous recovery theory posits that new learning is intrinsically volatile and spontaneously decays in a trial-by-trial basis (Scheidt et al., 2000; Smith et al., 2006; Criscimagna-Hemminger and Shadmehr, 2008; Galea et al., 2011; Brennan and Smith, 2015). Crucially, this theory stands on the assumption that EC trials disengage mechanisms of motor adaptation, since no positional error-signals are provided. Our findings challenge this notion since they suggest that availability of visual feedback regarding hand position during EC do in fact engage mechanisms of active motor adaptation.

We also found significant between-group differences when the state of the motor memory was tested after having washed-out the effect of the perturbation. When participants were re-exposed to EC trials (EC10) after a washout period (N2), the Force at Peak Velocity was significantly larger for the Augmented-Error group, when compared with both the Arc and Cursor groups. Pekny et al. (2011) reported that previous motor memories are retrieved when participants are exposed to increased levels of uncertainty—either by withholding reinforcement or applying random perturbations—at the onset of an EC block. Thus, higher levels on uncertainty for Arc group, due to the lack of visual feedback regarding movement trajectory, could lead to our results. However, we did not find differences between groups for the probability of success measured at EC10. Moreover, changes in the expression of the motor memory across time (i.e., Force at Peak Velocity measured at the end of EC9–EC10) were similar for all three groups, since differences observed at EC9 were maintained at EC10 (see Figure 12B). Thus, we suggest that re-exposure to the EC trials previously experienced led to the recall of the “adapted” motor memory for both experimental groups. This is in line with a motor adaptation model proposed by Lee and Schweighofer (2009), where a fast learning process with only one state works in parallel with a slow learning process with multiple states. Crucially, those authors suggested that activation of a specific slow state is determined by environmental conditions. When applied to our data, this model would predict that, when re-exposed to EC trials, the motor system would re-activate the slow state associated with that environmental context. This notion fits our results, since we found significantly greater Force at Peak Velocity at EC10 for the Augmented-Error group, when compared both with the Arc and Cursor groups, resembling the behavior found at the end of the previous EC block (EC9).

In conclusion, our results extend the notion that active learning occurs during the decay of the motor memory (Vaswani and Shadmehr, 2013; Vaswani et al., 2015). More specifically, our findings suggest an active involvement of motor adaptation mechanisms based on a mismatch between movement trajectory predicted by forward models, and actual sensory feedback.

## Author Contributions

AL-R designed the study, performed the data acquisition, analyzed the results, and wrote the article. RCM designed the study, analyzed the results, and wrote the article.

## Funding

This work was supported by the Wellcome Trust, grant WT087554.

## Conflict of Interest Statement

The authors declare that the research was conducted in the absence of any commercial or financial relationships that could be construed as a potential conflict of interest.
